# The prevention and response to infectious diseases in long-term care facilities in Korea: a nationwide survey

**DOI:** 10.4178/epih.e2024084

**Published:** 2024-10-17

**Authors:** Sun Hee Na, Joong Sik Eom, Sun Bean Kim, Hyung Jin Yoon, So Yeon Yoo, Kyeong Sook Cha, Jong Rim Choi, Ji Youn Choi, Si Hyeon Han, Jin Ju Park, Tark Kim, Jacob Lee

**Affiliations:** 1 Division of Infectious Disease, Department of Internal Medicine, Kangnam Sacred Heart Hospital, Hallym University College of Medicine, Seoul, Korea; 2 Division of Infectious Diseases, Department of Internal Medicine, Gil Medical Center, Gachon University College of Medicine, Incheon, Korea; 3 Division of Infectious Diseases, Department of Internal Medicine, Korea University College of Medicine, Seoul, Korea; 4 Department of Architecture, Dong Seoul University, Sungnam, Korea; 5 Gachon University College of Nursing, Incheon, Korea; 6 Department of Nursing Science, Sun Moon University, Asan, Korea; 7 Department of Nursing, Keimyung University Graduate School, Daegu, Korea; 8 Infection Control Office, Chung-Ang University Healthcare System, Seoul, Korea; 9 Infection Control Department, Dankook University Hospital, Cheonan, Korea; 10 Department of Internal Medicine, Soonchunhyang University Bucheon Hospital, Bucheon, Korea

**Keywords:** Long term care, Insurance, Surveys and questionnaires

## Abstract

**OBJECTIVES:**

Long-term care facilities (LTCFs) are communal environments for patients with chronic diseases or older adults, making them particularly susceptible to significant harm during infectious disease outbreaks. Nonetheless, LTCFs have historically been subject to less stringent infection prevention and control (IPC) mandates. This study aimed to assess the current state of LTCFs and to develop an IPC system tailored for these facilities following the coronavirus disease 2019 (COVID-19) pandemic.

**METHODS:**

We conducted an online survey of 11,366 LTCFs in Korea from December 30, 2022 to January 20, 2023, to evaluate the components of IPC in LTCFs. The infectious diseases targeted for IPC included COVID-19, influenza, and scabies. Additionally, we compared institution-based and home-based long-term care insurance facilities.

**RESULTS:**

Overall, 3,537 (31.1%) LTCFs responded to the survey, comprising 1,819 (51.4%) institution-based and 1,718 (48.6%) home-based facilities. A majority (87.4%, 2,376/2,720) of these facilities experienced COVID-19 outbreaks. However, only 42.2% of home-based facilities, in contrast to 90.6% of institution-based facilities, were equipped to manage concurrent COVID-19 cases. Similarly, while 92.1% of institution-based facilities were capable of managing influenza, only 50.5% of home-based facilities could do the same. The incidence of scabies was significantly higher in institution-based facilities than in home-based ones (26.1 vs. 4.3%). Additionally, 88.7% of institution-based facilities managed scabies cases effectively, compared to only 42.1% of home-based facilities.

**CONCLUSIONS:**

Approximately half of the LTCFs had a basic capacity to respond to infectious diseases. However, there were differences in response capabilities between institution-based facilities and home-based facilities.

## GRAPHICAL ABSTRACT

[Fig f1-epih-46-e2024084]

## Key Message

A survey of long-term care facilities (LTCFs) was conducted. About 50% of LTCFs had a basic capacity to respond to infectious diseases. However, there were differences in response capacity between institution-based and home-based facilities.

## INTRODUCTION

Residents of long-term care facilities (LTCFs) typically include older adults who suffer from chronic medical conditions, rendering them susceptible to infectious diseases [1-6]. During the coronavirus disease 2019 (COVID-19) pandemic, these older individuals experienced a higher mortality rate, with the risk of infection being particularly elevated in environments where they lived closely together [7-9]. Given the high prevalence of functional impairment among LTCF residents, frequent physical contact with healthcare workers is unavoidable. This characteristic complicates adherence to infection prevention and control (IPC) guidelines [10,11].

By August 2023, Korea had recorded a cumulative total of 34,572,554 confirmed COVID-19 cases and 35,605 deaths. There were 325,529 outbreaks of COVID-19 in long-term care hospitals (LTCHs) and LTCFs, resulting in 9,181 deaths [12]. A 2017 study found that 20.9% of LTCHs experienced influenza outbreaks among inpatients with infectious diseases [13]. Additionally, the incidence of scabies in LTCHs rose from 10% in 2014 to 58% in 2018, peaking at nearly 78% in 2017 [14].

In the United States, the Centers for Medicare and Medicaid Services (CMS) oversee LTCFs to ensure they meet established standards. In 2016, CMS introduced an IPC program for LTCFs, which included the appointment of 1 or more infection prevention officers. This program was to be fully implemented by 2019 [15]. In Korea, under Article 47 of the Medical Service Act, hospitals are required to employ dedicated IPC staff. The requirement for dedicated IPC staff has been progressively enforced and is now mandatory in hospitals with 100 or more beds [16].

Despite representing a high-risk setting for infections, LTCFs are not classified as healthcare facilities and, until recently, were highly vulnerable to outbreaks due to a lack of government regulations on IPC. In 2021 and 2023, the National Health Insurance Service (NHIS) developed and distributed infection control manuals for LTCFs in 2 phases. However, these manuals were only recommended for reference and were not mandatory. Unlike LTCHs, which have mandatory guidelines for staffing and training in IPC, LTCFs lack specific guidelines. Since the COVID-19 pandemic, there has been increased attention to IPC in LTCFs. A 2022 survey conducted by the Korea Disease Control and Prevention Agency of LTCHs and LTCFs revealed that 60% of the institutions had dedicated IPC staff, and over 95% possessed an infectious disease manual [17]. Despite these measures, COVID-19 outbreaks in LTCHs and LTCFs remained significant, accounting for about a quarter of the deaths. There is a notable lack of data on the status of infectious disease responses in LTCFs in Korea.

This study focuses on the survey results related to the prevention and response to infectious diseases in LTCFs. Its aim was to evaluate the current situation in these facilities and to develop a tailored IPC system for them following the COVID-19 pandemic.

## MATERIALS AND METHODS

### Study design

We conducted an online survey of 11,366 LTCFs in Korea from December 30, 2022, to January 20, 2023. According to the 2022 Office for National Statistics, the country hosts 5,090 day and night care facilities with a capacity for 177,842 individuals, 126 short-term respite care facilities accommodating 1,146 individuals, 4,372 long-term care insurance (LTCI) facilities serving 218,737 individuals, and 1,778 community-based LTCI homes for 15,707 individuals. The NHIS, Korea’s sole centralized public insurer, administers the LTCI [18]. LTCI benefits are categorized as home-based, institution-based, and special cash benefits under Article 23 of the Long-Term Care Insurance Act in Korea [19]. LTCFs are classified according to the type of LTCI benefits they provide, into home-based and institution-based facilities. Home-based facilities offer services such as home visit care, home visit bathing, home visit nursing, day and night care, and short-term respite care. The study focused on 4 types of LTCFs: day and night care facilities, short-term respite care in home-based facilities, LTCI facilities, and community-based LTCI homes in institution-based facilities.

### Questionnaire development

Six infectious disease physicians and 5 IPC nurses developed the questionnaire. The survey questionnaire was modeled on the Infection Control Assessment and Response (ICAR) tool [20], which was published by the US Centers for Disease Control and Prevention (CDC) and adapted for Korean conditions. The ICAR tool is designed to evaluate IPC practices across various healthcare settings and to facilitate quality improvement initiatives.

The survey included questions about the respondent’s LTCF type, the respondent’s position, their years of experience, geographic region, the nature of the building or housing facility, its capacity, the various staff types, and any affiliated doctors or hospitals. The questionnaire also addressed components related to the prevention and management of infectious diseases in LTCFs.

The infectious diseases targeted in this study included COVID-19, influenza, and scabies. Influenza and scabies are the most prevalent epidemic infections among LTCF residents [21-24]. Since the onset of the 2020 pandemic, managing COVID-19 in LTCFs has posed significant challenges. The Nursing Home COVID-19 ICAR tool [25], developed during the COVID-19 pandemic, served as the basis for the COVID-19 questionnaire. The questions were finalized in consultation with the NHIS. Following the final revisions, the survey was distributed online to national LTCFs. SurveyMonkey (SurveyMonkey, San Mateo, CA, USA; http://www. surveymonkey.com) was utilized for the online survey platform. Respondents accessed the computerized online system and entered their responses using a self-completion method.

### Statistical analysis

The study analyzed data categorized by insurance type, presenting continuous variables as means with standard deviations or as medians with interquartile ranges. Dichotomous data were reported as counts and percentages. Quantitative data comparisons were conducted using the Student t-test or the Mann-Whitney U test, whereas qualitative data were analyzed using the Pearson chi-square test. A p-value of less than 0.05 was considered statistically significant. All statistical analyses were carried out using SPSS version 28.0 (IBM Corp., Armonk, NY, USA).

### Ethics statement

This study was reviewed and approved by the Institutional Review Board of Hallym University Kangnam Sacred Heart Hospital (2022-09-022).

## RESULTS

### Characteristics of respondents

Altogether, 3,537 of the 11,366 LTCFs (31.1%) responded to the online survey. Of these, 1,819 were institution-based facilities, with a 29.6% response rate, compared to a 32.9% response rate from 1,718 home-based facilities (p<0.001). The respondents included 1,710 (48.3%) day and night care facilities, 1,436 (40.6%) LTCI facilities, 383 (10.8%) community-based LTCI homes, and 8 (0.2%) short-term respite care facilities. Among the institution-based facilities, 807 (44.4%) were located in Seoul, Gyeonggi, and Incheon; 366 (20.1%) in Gyeongsang; 276 (15.2%) in Chungcheong; 272 (15.0%) in Jeolla and Jeju; and 98 (5.4%) in Gangwon. For home-based facilities, 592 (34.5%) were in Seoul, Gyeonggi, and Incheon; 518 (30.2%) in Gyeongsang; 289 (16.8%) in Jeolla and Jeju; 268 (15.6%) in Chungcheong; and 51 (3.0%) in Gangwon (p<0.001). The questionnaires were primarily completed by facility chiefs or social workers. The work experience of the respondents ranged from less than 1 year to more than 5 years, with approximately a quarter having less than 1 year of experience (24.4 vs. 23.0%) (Table 1).

### Characteristics of facilities

Institution-based facilities primarily utilized detached buildings (69.2%), while home-based facilities predominantly occupied attached buildings (61.2%). The survey also included larger facilities, with 5.4% of institution-based facilities accommodating 100 or more individuals. Additionally, over 20% of both institution-based and home-based facilities had a capacity for 50 or more people (28.2 vs. 20.7%). The proportion of residents requiring special care, such as those with urinary tract catheters, nasoesophageal feeding tubes, bedsores, or needing injections, was significantly higher in institution-based facilities compared to home-based ones. Institution-based facilities frequently contracted with both hospitals and doctors, whereas home-based facilities typically contracted only with hospitals, and 27.8% of these had no such contracts.

### Response to coronavirus disease 2019, influenza, and scabies

The response rates for each infectious disease were as follows: 76.9% (2,720/3,538) for COVID-19, 76.2% (2,697/3,538) for influenza, and 75.7% (2,678/3,538) for scabies. Most facilities had an outbreak of COVID-19 (91.2 vs. 84.3%). However, widespread outbreaks (affecting > 30% of the population) primarily occurred in institution-based facilities. Both facility types had response systems for COVID-19. Most respondents answered that the staff knew how to manage COVID-19 patients and exposed individuals. Rapid antigen tests (RATs) were used in more than 90% of facilities where COVID-19 testing was required. Only 42% of home-based facilities were confident in their ability to manage COVID-19 cases, in contrast to 90.6% of institution-based facilities. A higher percentage of institution-based facilities were able to isolate confirmed cases compared to home-based facilities (91.7 vs. 53.7%) (Table 2).

In contrast to COVID-19, fewer than 20% of facilities reported an outbreak of influenza. When influenza testing was required, 90.8% of institution-based facilities and 67.2% of home-based facilities sought testing at external hospitals. In 28.8% of home-based facilities, suspected patients were discharged without undergoing testing. Similar to the situation with COVID-19, 92.1% of institution-based facilities felt confident in their ability to manage influenza cases, in contrast to only 50.5% of home-based facilities. Over 95% of the facilities either coordinated or recommended influenza vaccinations directly (Table 3).

The incidence of scabies was significantly higher in institution-based facilities compared to home-based facilities (26.1 vs. 4.3%). When scabies testing was mandated within the facility, the most common course of action was to refer patients to an outside hospital for diagnosis, resulting in 42.7% of patients from home-based facilities being discharged. Confirmed cases were managed in 88.7% of the institution-based facilities, in contrast to only 42.1% of the home-based facilities (Table 4).

COVID-19 response manuals were available in over 95% of both types of facilities. Manuals for influenza and scabies were less commonly held compared to those for COVID-19. Home-based facilities were less likely to include guidelines on quarantine and management of confirmed cases in their manuals (Table 5).

We also compared LTCFs divided into 4 types: day and night care facilities, short-term respite care in home-based facilities, LTCI facilities, and community-based LTCI homes in institution-based facilities. This analysis yielded results similar to those observed for the type of LTCI benefit (Supplementary Materials 1-5).

## DISCUSSION

This study involved an extensive nationwide survey, covering nearly one-third of the LTCFs in Korea. Although LTCFs are not categorized as medical facilities, our research confirmed that infectious diseases, including influenza, scabies, and COVID-19, are prevalent within these settings. A significant number of the facilities have established a basic response system for COVID-19; however, the measures for controlling influenza and scabies appear to be comparatively neglected. Moreover, home-based facilities are less equipped with resources for managing infectious diseases compared to institution-based facilities. These findings will provide a crucial foundation for the development of future IPC policies for LTCFs in Korea.

Our survey had a response rate of 31.1% from all LTCFs, with 29.6% from institution-based facilities and 32.9% from home-based facilities. The survey was completed by facilities nationwide. This response rate is reasonable compared to typical survey response rates. However, there is room for improvement to achieve more accurate results. One strategy to enhance response rates could be to make regular surveys mandatory.

The proportion of residents requiring special care, such as those with urinary tract catheters, nasoesophageal feeding tubes, bedsores, and those needing injections, was higher in institution-based facilities. We observed that medical care was frequently necessary in LTCFs, which primarily facilitated the transfer of patients to and from acute care and LTCHs. The IPC systems of acute care hospitals, LTCHs, and LTCFs are interdependent [26,27]. However, the medical sector is often overseen by affiliated doctors or hospitals, which complicates their ability to actively influence IPC practices in LTCFs. This factor is crucial in the design of IPC systems.

The COVID-19 pandemic significantly impacted numerous LTCFs [7-9]. According to a survey, an outbreak occurred in 87.4% of the responding facilities. Following their experiences during the pandemic, over 90% of these facilities have established a COVID-19 response system, and their staff are now knowledgeable in handling such crises. Additionally, it was feasible to isolate and manage both staff and confirmed cases within the LTCFs. This indicates that the experience gained in responding to COVID-19 could greatly improve the ability of LTCFs to respond to new infectious disease outbreaks.

There were fewer cases of influenza and scabies than COVID-19. Like severe acute respiratory syndrome coronavirus 2 (SARS-CoV-2), influenza can be tested using the RAT, but most diagnoses occur outside of hospital settings. Similarly, the diagnosis of scabies is entirely dependent on facilities outside hospitals. If staff caring for residents are not aware of the potential for infectious diseases and do not seek diagnosis in an external hospital, cases may go undiagnosed. Consequently, the incidence of infectious diseases in LTCFs may be underestimated [24].

Home-based facilities generally exhibit significantly lower management capacities, despite having tests, manuals, and response systems in place. This discrepancy becomes apparent when comparing the manual components. For instance, while 36.8% of institution-based facilities have affiliated doctors, only 1.0% of home-based facilities do, and even when including hospitals, 27.8% of home-based facilities lack affiliated doctors or hospitals. In home-based settings, diagnosing and managing patients before they are transferred to a hospital often poses challenges. This difficulty extends to managing IPC issues when they arise. Additionally, there is a notable disparity in the proportion of patients requiring special medical care—such as those with urinary tract catheters, nasoesophageal tubes, bedsores, and injections—between the 2 types of facilities. The ability to manage these patients effectively is what distinguishes these facilities.

Unlike institution-based facilities, which are paid a flat rate per day for each admitted resident, home-based facilities are compensated based on the duration and frequency of services used. Additionally, the maximum monthly benefits limit for a resident under this payment system is lower for home-based facilities than for institution-based benefit insurance [28]. IPC is inherently less robust in home-based facilities compared to institution-based ones. This disparity should be taken into account when developing guidelines for responding to infectious diseases.

There is a need to develop and distribute a manual that reflects the differences between these 2 facilities and can be applied in real situations. Once implemented, the manual should be periodically reviewed to ensure its appropriate application. Additionally, training is necessary to ensure its correct execution.

Our study had several limitations. First, there is a possibility that some respondents did not fully understand the status of the facility or its IPC program. Notably, 23.7% of the respondents had been employed for less than a year, potentially leading to inaccuracies in their responses due to a lack of familiarity with the current IPC situation at the facility. Second, the capacity of LTCFs to respond to infectious diseases might have been overestimated due to selection bias. This bias could stem from the fact that LTCFs that were relatively well-prepared for handling infectious diseases were more likely to participate in the survey, thus skewing the results. Third, our analysis was based on data from the respondents’ answers. Over time, respondents may have altered their memories or provided overly positive responses due to concerns about potential consequences.

In conclusion, infectious disease outbreaks are common in LTCFs, which generally possess the basic capacity to respond to them. However, when categorized by institution-based and home-based benefits, variations in their response capabilities to infectious diseases become apparent. This indicates a need for guidelines, support, and systems that are specifically tailored to the type of LTCF. Consequently, it is essential to conduct periodic studies that survey and analyze the management of infectious diseases beyond COVID-19, influenza, and scabies. Such surveys should be carried out nationally or, at a minimum, at the municipal level.

## Figures and Tables

**Figure f1-epih-46-e2024084:**
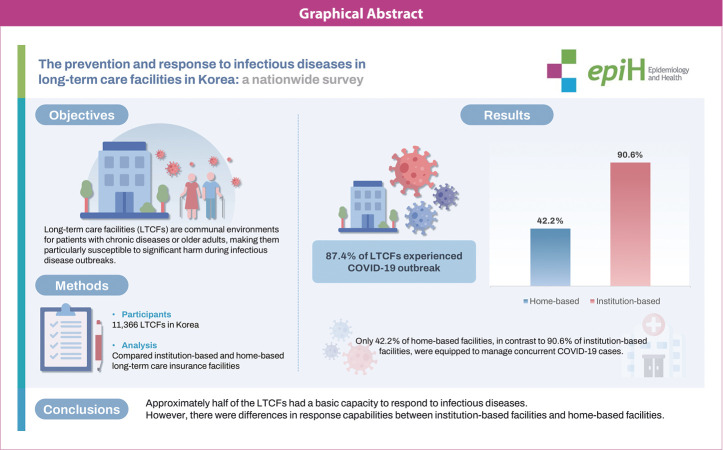


**Table 1.** Characteristics of the respondents and facilities

Characteristics	Institution-based (n=1,819)	Home-based (n=1,718)	p-value
Respondent position			<0.001
Facility chief	648/1,819 (35.6)	685/1,718 (39.9)	
Office director	257/1,819 (14.1)	50/1,718 (2.9)	
Social worker	707/1,819 (38.9)	744/1,718 (43.3)	
Nurse	74/1,819 (4.1)	55/1,718 (3.2)	
Nursing assistant	133/1,819 (7.3)	184/1,718 (10.7)	
Years of experience (yr)			<0.001
<1	418/1,819 (23.0)	419/1,718 (24.4)	
1-2	322/1,819 (17.7)	307/1,718 (17.9)	
2-5	472/1,819 (25.9)	649/1,718 (37.8)	
>5	607/1,819 (33.4)	343/1,718 (20.0)	
Age of responder	47.4±11.2	46.6±10.6	0.031
Type of building or housing facility			<0.001
An independent building	1,258/1,819 (69.2)	666/1,718 (38.8)	
Location in building, separate paths	337/1,819 (18.5)	651/1,718 (37.9)	
Location in building, no separate paths	224/1,819 (12.3)	401/1,718 (23.3)	
Capacity (person)			<0.001
<10	386/1,817 (21.2)	72/1,716 (4.2)	
10-29	628/1,817 (34.6)	549/1,716 (32.0)	
30-49	290/1,817 (16.0)	739/1,716 (43.1)	
50-99	414/1,817 (22.8)	340/1,716 (19.8)	
≥100	99/1,817 (5.4)	16/1,716 (0.9)	
Residents with special care needs			
Urinary tract catheters	213/1,819 (11.7)	11/1,718 (0.6)	<0.001
Nasoesophageal feeding tubes	375/1,819 (20.6)	7/1,718 (0.4)	<0.001
Bedsores	135/1,819 (7.4)	35/1,718 (2.0)	<0.001
Injections (IV or IM)	36/1,819 (2.0)	9/1,718 (0.5)	<0.001
Affiliated doctors or hospitals			<0.001
Neither	27/1,819 (1.5)	477/1,718 (27.8)	
Doctors only	670/1,819 (36.8)	18/1,718 (1.0)	
Hospitals only	342/1,819 (18.8)	1,195/1,718 (69.6)	
Both	780/1,819 (42.9)	28/1,718 (1.6)	

Values are presented as number (%) or mean±standard deviation. IV, intravenous; IM, intramuscular.

**Table 2.** Comparison of prevention and response to COVID-19 between institution-based and home-based facilities

Variables	Institution-based (n=1,819)	Home-based (n=1,718)	p-value
COVID-19 outbreak in facility			<0.001
Widespread outbreak	1,002/1,422 (70.5)	558/1,298 (43.0)	
Partial outbreak	280/1,422 (19.7)	536/1,298 (41.3)	
COVID-19 response system in facility	1,380/1,422 (97.0)	1,233/1,298 (95.0)	0.006
COVID-19 response system worked in practice	910/1,043 (87.2)	915/978 (93.6)	<0.001
Staff know information about each case	1,401/1,413 (99.2)	1,276/1,284 (99.4)	0.494
Staff know classification of exposure cases	1,409/1,413 (99.7)	1,273/1,284 (99.1)	0.045
COVID-19 test			<0.001
RAT in facility	1,275/1,412 (90.3)	1,170/1,284 (91.1)	
Outside hospitals	137/1,412 (9.7)	95/1,284 (7.4)	
Discharge	0/1,412 (0.0)	19/1,284 (1.5)	
Facility can manage COVID-19 cases	1,280/1,413 (90.6)	542/1,284 (42.2)	<0.001
Facility can isolate confirmed and exposed cases			<0.001
Confirmed and exposed cases	433/1,413 (30.6)	325/1,284 (25.3)	
Only confirmed cases	861/1,413 (60.9)	767/1,284 (28.4)	
Facility can isolate staff and confirmed patients	1,243/1,413 (88.0)	1,102/1,284 (85.8)	0.099

Values are presented as number (%). COVID-19, coronavirus disease 2019; RAT, rapid antigen test.

**Table 3.** Comparison of prevention and response to influenza between institution-based and home-based facilities

Variables	Institution-based (n=1,819)	Home-based (n=1,718)	p-value
Cases of influenza in facility	246/1,413 (17.4)	204/1,284 (15.9)	0.290
Influenza test			<0.001
Outside hospitals	1,273/1,402 (90.8)	858/1,276 (67.2)	
Discharge	61/1,402 (4.4)	367/1,276 (28.8)	
No guidance	68/1,402 (4.9)	51/1,276 (4.0)	
Facility can manage influenza cases	1,291/1,402 (92.1)	645/1,276 (50.5)	<0.001
Influenza vaccination			<0.001
Vaccinated with facility intervention	1,264/1,402 (90.2)	997/1,276 (78.1)	
Vaccination recommended	79/1,402 (5.6)	252/1,276 (19.7)	
No guidance	59/1,402 (4.2)	27/1,276 (2.1)	

Values are presented as number (%).

**Table 4.** Comparison of prevention and response to scabies between institution-based and home-based facilities

Variables	Institution-based (n=1,819)	Home-based (n=1,718)	p-value
Cases of scabies in facility	366/1,402 (26.1)	55/1,276 (4.3)	<0.001
Scabies test			<0.001
Outside hospitals	1,296/1,401 (92.5)	729/1,273 (57.3)	
Discharge	58/1,401 (4.1)	382/1,273 (30.0)	
No guidance	47/1,401 (3.4)	162/1,273 (12.7)	
Facility can manage scabies cases	1,242/1,401 (88.7)	536/1,273 (42.1)	<0.001

Values are presented as number (%).

**Table 5.** Components of the coronavirus disease 2019 (COVID-19), influenza, and scabies manuals

Components	Institution-based (n=1,819)	Home-based (n=1,718)	p-value
COVID-19 manual	1,394/1,421 (98.1)	1,248/1,296 (96.3)	0.004
How to report	1,314/1,394 (94.3)	1,054/1,248 (84.5)	
Management of suspect patients	1,360/1,394 (97.6)	1,189/1,248 (95.3)	
Management of exposed patients	1,305/1,394 (93.6)	1,111/1,248 (89.0)	
Management of exposed staff	1,302/1,394 (93.4)	1,092/1,248 (87.5)	
Movement and quarantine within facility	1,236/1,394 (88.7)	951/1,248 (76.2)	
Management of confirmed patients	1,266/1,394 (90.8)	731/1,248 (58.6)	
Influenza manual	1,002/1,413 (70.9)	783/1,284 (61.0)	<0.001
How to report	808/1,002 (80.6)	574/783 (73.3)	
Management of suspect patients	931/1,002 (92.9)	717/783 (91.6)	
Management of exposed patients	874/1,002 (87.2)	634/783 (81.0)	
Management of exposed staff	823/1,002 (82.1)	615/783 (78.5)	
Quarantine within facility	843/1,002 (84.1)	568/783 (72.5)	
Management of confirmed patients	815/1,002 (81.3)	461/783 (58.9)	
Scabies manual	1,114/1,402 (79.5)	651/1,276 (51.0)	<0.001
How to report	971/1,114 (87.2)	532/651 (81.7)	
Management of suspect patients	1,048/1,114 (94.1)	585/651 (89.9)	
Management of exposed patients	1,009/1,114 (90.6)	559/651 (85.9)	
Management of exposed staff	973/1,114 (87.3)	540/651 (82.9)	
Quarantine within facility	1,032/1,114 (92.6)	511/651 (78.5)	
Management of confirmed patients	978/1,114 (87.8)	413/651 (63.4)	

Values are presented as number (%).

## Supplementary materials

Supplementary Material 1. Respondents and facilities characteristics

Supplementary Material 2. Comparison of prevention and response to COVID-19 with institution-based and home-based facilities

Supplementary Material 3. Comparison of prevention and response to influenza with institution-based and home-based facilities

Supplementary Material 4. Comparison of prevention and response to scabies with institution-based and home-based facilities

Supplementary Material 5. Components of the COVID-19, influenza, and scabies manual
