# Supplementary material for: The prevention and response to infectious diseases in long-term care facilities in Korea: a nationwide survey
Source: Epidemiol Health. 2024 Oct 17;46:e2024084. doi: 10.4178/epih.e2024084 (PMC11832240; doi:10.4178/epih.e2024084)
Supplement: Supplementary Material 1. — Respondents and facilities characteristics [file epih-46-e2024084-Supplementary-1.docx]

**Supplementary Material 1.** Respondents and facilities characteristics

|  | Community-based LTCI homes  (n = 383) | LTCI facilities  (n = 1436) | Day and night care facilities  (n = 1710) | Short-term respite care (n = 8) | *P*-value |
| --- | --- | --- | --- | --- | --- |
| Respondent position |  |  |  |  | <0.001 |
| Facility chief | 257/383 (67.1%) | 391/1436 (27.2%) | 681/1710 (39.8%) | 4/8 (50.0%) |  |
| Office director | 3/383 (0.8%) | 254/1436 (17.7%) | 50/1710 (2.9%) | 0/8 (0.0%) |  |
| Social worker | 75/383 (19.6%) | 632/1436 (44.0%) | 741/1710 (43.3%) | 3/8 (37.5%) |  |
| Nurse | 3/383 (0.8%) | 71/1436 (4.9%) | 55/1710 (3.2%) | 0/8 (0.0%) |  |
| Nursing assistant | 45/383 (11.7%) | 88/1436 (6.1%) | 183/1710 (10.7%) | 1/8 (12.5%) |  |
| Years of experience |  |  |  |  | <0.001 |
| Less than 1 year | 84/383 (21.9%) | 334/1436 (23.3%) | 418/1710 (24.4%) | 1/8 (12.5%) |  |
| 1-2 years | 65/383 (17.0%) | 257/1436 (17.9%) | 306/1710 (17.9%) | 1/8 (12.5%) |  |
| 2-5 years | 98/383 (25.6%) | 374/1436 (26.0%) | 647/1710 (37.8%) | 2/8 (25.0%) |  |
| More than 5 years | 136/383 (35.5%) | 471/1436 (32.8%) | 339/1710 (19.8%) | 4/8 (50.0%) |  |
| Age of responder (year) | 50.3 ± 11.3 | 46.6 ± 11.0 | 47.5 ± 10.6 | 51.5 ± 12.8 | <0.001 |
| Type of building or housing facility |  |  |  |  | <0.001 |
| Use an independent building | 203/383 (53.0%) | 1055/1436 (73.5%) | 664/1710 (38.8%) | 2/8 (25.0%) |  |
| Location in building, separate paths | 109/383 (28.5%) | 228/1436 (15.9%) | 646/1710 (37.8%) | 5/8 (62.5%) |  |
| Location in building, no separate paths | 71/383 (18.5%) | 153/1436 (10.7%) | 400/1710 (23.4%) | 1/8 (12.5%) |  |
| Capacity |  |  |  |  | 0.000 |
| Less than 10 persons | 371/383 (96.9%) | 15/1436 (1.0%) | 67/1710 (3.9%) | 5/8 (62.5%) |  |
| 10-29 persons | 11/383 (2.9%) | 617/1436 (43.0%) | 548/1710 (32.1%) | 1/8 (12.5%) |  |
| 30-49 persons | 0/383 (0.0%) | 290/1436 (20.2%) | 737/1710 (43.1%) | 2/8 (25.0%) |  |
| 50-99 persons | 1/383 (0.3%) | 413/1436 (28.8%) | 340/1710 (19.9%) | 0/8 (0.0%) |  |
| More than 100 persons | 0/383 (0.0%) | 99/1436 (6.9%) | 16/1710 (0.9%) | 0/8 (0.0%) |  |
| Residents with special care needs |  |  |  |  |  |
| Urinary tract catheters | 53/383 (13.8%) | 160/1436 (11.1%) | 11/1710 (0.6%) | 0/8 (0.0%) | <0.001 |
| Nasoesophageal feeding tubes | 57/383 (14.9%) | 318/1436 (22.1%) | 6/1710 (0.4%) | 1/8 (12.5%) | <0.001 |
| Bedsores | 28/383 (7.3%) | 107/1436 (7.5%) | 34/1710 (2.0%) | 1/8 (12.5%) | <0.001 |
| Injections (IV or IM) | 8/383 (2.1%) | 28/1436 (1.9%) | 9/1710 (0.5%) | 0/8 (0.0%) | 0.002 |
| Affiliated doctors or hospitals |  |  |  |  | 0.000 |
| Neither | 13/383 (3.4%) | 14/1436 (1.0%) | 474/1710 (27.7%) | 3/8 (37.5%) |  |
| Doctors only | 115/383 (30.0%) | 555/1436 (38.6%) | 17/1710 (1.0%) | 1/8 (12.5%) |  |
| Hospitals only | 123/383 (32.1%) | 219/1436 (15.3%) | 1192/1710 (69.7%) | 3/8 (37.5%) |  |
| Both | 132/383 (34.5%) | 648/1436 (45.1%) | 27/1710 (1.6%) | 1/8 (12.5%) |  |

**Note.** IV: Intravenous; IM: Intramuscular

The data indicate the number (%) or mean value ± standard deviation.
