# Supplementary material for: The prevention and response to infectious diseases in long-term care facilities in Korea: a nationwide survey
Source: Epidemiol Health. 2024 Oct 17;46:e2024084. doi: 10.4178/epih.e2024084 (PMC11832240; doi:10.4178/epih.e2024084)
Supplement: Supplementary Material 2. — Comparison of prevention and response to COVID-19 with institution-based and home-based facilities [file epih-46-e2024084-Supplementary-2.docx]

**Supplementary Material 2.** Comparison of prevention and response to COVID-19 with institution-based and home-based facilities

|  | Community-based LTCI homes  (n = 383) | LTCI facilities  (n = 1436) | Day and night care facilities  (n = 1710) | Short-term respite care (n = 8) | *P*-value |
| --- | --- | --- | --- | --- | --- |
| COVID-19 outbreak in facility |  |  |  |  | <0.001 |
| Widespread outbreak | 198/307 (64.5%) | 804/1115 (72.1%) | 555/1293 (42.9%) | 3/5 (60.0%) |  |
| Partial outbreak | 42/307 (13.7%) | 238/1115 (21.3%) | 536/1293 (41.5%) | 0/5 (0.0%) |  |
| COVID-19 response system in facility | 291/307 (94.8%) | 1089/1115 (97.7%) | 1228/1293 (95.0%) | 5/5 (100%) | 0.004 |
| COVID-19 response system worked in practice | 175/201 (87.1%) | 735/842 (87.3%) | 913/976 (93.5%) | 2/2 (100%) | <0.001 |
| Staff know information in the case | 304/305 (99.7%) | 1097/1108 (99.0%) | 1272/1280 (99.4%) | 4/4 (100%) | 0.588 |
| Staff know classification of exposure cases | 305/305 (100%) | 1104/1108 (99.6%) | 1269/1280 (99.1%) | 4/4 (100%) | 0.202 |
| COVID-19 test |  |  |  |  | <0.001 |
| RAT in facility | 270/304 (88.8%) | 1005/1108 (90.7%) | 1166/1280 (91.1%) | 4/4 (100%) |  |
| Outside hospitals | 34/304 (11.2%) | 103/1108 (9.3%) | 95/1280 (7.4%) | 0/4 (0.0%) |  |
| Discharge | 0/304 (0.0%) | 0/1108 (0.0%) | 19/1280 (1.5%) | 0/4 (0.0%) |  |
| Facility can manage COVID-19 cases | 259/305 (84.9%) | 1021/1108 (92.1%) | 539/1280 (42.1%) | 3/4 (75.0%) | <0.001 |
| Facility can isolate confirmed and exposed cases |  |  |  |  | <0.001 |
| Confirmed and exposed cases | 68/305 (22.3%) | 365/1108 (32.9%) | 323/1280 (25.2%) | 2/4 (50.0%) |  |
| Only confirmed cases | 192/305 (63.0%) | 669/1108 (60.4%) | 766/1280 (59.8%) | 1/4 (25.0%) |  |
| Facility can isolate staff and confirmed patients | 249/305 (81.6%) | 994/1108 (89.7%) | 1098/1280 (85.8%) | 4/4 (100%) | <0.001 |

**Note.** RAT: Rapid antigen test

The data indicate the number (%).
