# Supplementary material for: The prevention and response to infectious diseases in long-term care facilities in Korea: a nationwide survey
Source: Epidemiol Health. 2024 Oct 17;46:e2024084. doi: 10.4178/epih.e2024084 (PMC11832240; doi:10.4178/epih.e2024084)
Supplement: Supplementary Material 3. — Comparison of prevention and response to influenza with institution-based and home-based facilities [file epih-46-e2024084-Supplementary-3.docx]

**Supplementary Material 3.** Comparison of prevention and response to influenza with institution-based and home-based facilities

|  | Community-based LTCI homes  (n = 383) | LTCI facilities  (n = 1436) | Day and night care facilities  (n = 1710) | Short-term respite care (n = 8) | *P*-value |
| --- | --- | --- | --- | --- | --- |
| Cases of influenza in facility | 38/305 (12.5%) | 208/1108 (18.8%) | 203/1280 (15.9%) | 1/4 (25.0%) | 0.042 |
| Influenza test |  |  |  |  | <0.001 |
| Outside hospitals | 275/302 (91.1%) | 998/1100 (90.7%) | 854/1272 (67.1%) | 4/4 (100%) |  |
| Discharge | 10/302 (3.3%) | 51/1100 (4.6%) | 367/1272 (28.9%) | 0/4 (0.0%) |  |
| No guidance | 17/302 (5.6%) | 51/1100 (4.6%) | 51/1272 (4.0%) | 0/4 (0.0%) |  |
| Facility can manage influenza cases | 259/302 (85.8%) | 1032/1100 (93.8%) | 642/1272 (50.5%) | 3/4 (75.0%) | <0.001 |
| Influenza vaccination |  |  |  |  | <0.001 |
| Vaccinated with facility intervention | 270/302 (89.4%) | 994/1100 (90.4%) | 993/1272 (78.1%) | 4/4 (100%) |  |
| Vaccination recommended | 18/302 (6.0%) | 61/1100 (18.4%) | 252/1272 (19.8%) | 0/4 (0.0%) |  |
| No guidance | 14/302 (4.6%) | 45/1100 (4.1%) | 27/1272 (2.1%) | 0/4 (0.0%) |  |

The data indicate the number (%)
