# Supplementary material for: The prevention and response to infectious diseases in long-term care facilities in Korea: a nationwide survey
Source: Epidemiol Health. 2024 Oct 17;46:e2024084. doi: 10.4178/epih.e2024084 (PMC11832240; doi:10.4178/epih.e2024084)
Supplement: Supplementary Material 4. — Comparison of prevention and response to scabies with institution-based and home-based facilities [file epih-46-e2024084-Supplementary-4.docx]

**Supplementary Material 4.** Comparison of prevention and response to scabies with institution-based and home-based facilities

|  | Community-based LTCI homes  (n = 383) | LTCI facilities  (n = 1436) | Day and night care facilities  (n = 1710) | Short-term respite care (n = 8) | *P*-value |
| --- | --- | --- | --- | --- | --- |
| Cases of scabies in facility | 29/302 (9.6%) | 337/1100 (30.6%) | 55/1272 (4.3%) | 0/4 (0.0%) | <0.001 |
| Scabies test |  |  |  |  | <0.001 |
| Outside hospitals | 275/301 (91.4%) | 1021/1100 (92.8%) | 726/1269 (57.2%) | 3/4 (75.0%) |  |
| Discharge | 13/301 (4.3%) | 45/1100 (4.1%) | 382/1269 (30.1%) | 0/4 (0.0%) |  |
| No guidance | 13/301 (4.3%) | 34/1100 (3.1%) | 161/1269 (12.7%) | 1/4 (25.0%) |  |
| Facility can manage scabies cases | 247/301 (82.1%) | 995/1100 (90.5%) | 533/1269 (42.0%) | 3/4 (75.0%) | <0.001 |

The data indicate the number (%)
