# Supplementary material for: The prevention and response to infectious diseases in long-term care facilities in Korea: a nationwide survey
Source: Epidemiol Health. 2024 Oct 17;46:e2024084. doi: 10.4178/epih.e2024084 (PMC11832240; doi:10.4178/epih.e2024084)
Supplement: Supplementary Material 5. — Components of the COVID-19, influenza, and scabies manual [file epih-46-e2024084-Supplementary-5.docx]

**Supplementary Material 5.** Components of the COVID-19, influenza, and scabies manual

|  | Community-based LTCI homes  (n = 383) | LTCI facilities  (n = 1436) | Day and night care facilities  (n = 1710) | Short-term respite care (n = 8) | *P*-value |
| --- | --- | --- | --- | --- | --- |
| COVID-19 manual | 300/307 (97.7%) | 1094/1114 (98.2%) | 1244/1292 (96.3%) | 4/4 (100%) | 0.035 |
| How to report | 279/300 (93.0%) | 1035/1094 (94.6%) | 1050/1244 (84.4%) | 4/4 (100%) |  |
| Management of suspect patients | 292/300 (97.3%) | 1068/1094 (97.6%) | 1185/1244 (95.3%) | 4/4 (100%) |  |
| Management of exposed patients | 279/300 (93.0%) | 1026/1094 (93.8%) | 1108/1244 (89.1%) | 3/4 (75.0%) |  |
| Management of exposed staff | 282/300 (94.0%) | 1020/1094 (93.2%) | 1089/1244 (87.5%) | 3/4 (75.0%) |  |
| Movement and quarantine within facility | 253/300 (84.3%) | 983/1094 (89.9%) | 948/1244 (76.2%) | 3/4 (75.0%) |  |
| Management of confirmed patients | 271/300 (90.3%) | 995/1094 (91.0%) | 727/1244 (58.4%) | 4/4 (100%) |  |
| Influenza manual | 222/305 (72.8%) | 780/1108 (70.4%) | 779/1280 (60.9%) | 4/4 (100%) | <0.001 |
| How to report | 176/222 (79.3%) | 632/780 (81.0%) | 570/779 (73.2%) | 4/4 (100%) |  |
| Management of suspect patients | 211/222 (95.0%) | 720/780 (92.3%) | 713/779 (91.5%) | 4/4 (100%) |  |
| Management of exposed patients | 200/222 (90.1%) | 674/780 (86.4%) | 632/779 (81.1%) | 2/4 (50.0%) |  |
| Management of exposed staff | 184/222 (82.9%) | 639/780 (81.9%) | 613/779 (78.7%) | 2/4 (50.0%) |  |
| Quarantine within facility | 185/222 (83.3%) | 658/780 (84.4%) | 566/779 (72.7%) | 2/4 (50.0%) |  |
| Management of confirmed patients | 179/222 (80.6%) | 636/780 (81.5%) | 459/779 (58.9%) | 2/4 (50.0%) |  |
| Scabies manual | 237/302 (78.5%) | 877/1100 (79.7%) | 649/1272 (51.0%) | 2/4 (50.0%) | <0.001 |
| How to report | 205/237 (86.5%) | 766/877 (87.3%) | 530/649 (81.7%) | 2/2 (100%) |  |
| Management of suspect patients | 220/237 (92.8%) | 828/877 (94.4%) | 583/649 (89.8%) | 2/2 (100%) |  |
| Management of exposed patients | 217/237 (91.6%) | 792/877 (90.3%) | 557/649 (85.8%) | 2/2 (100%) |  |
| Management of exposed staff | 203/237 (85.7%) | 770/877 (87.8%) | 538/649 (82.9%) | 2/2 (100%) |  |
| Quarantine within facility | 221/237 (93.2%) | 811/877 (92.5%) | 509/649 (78.4%) | 2/2 (100%) |  |
| Management of confirmed patients | 203/237 (85.7%) | 775/877 (88.4%) | 411/649 (63.3%) | 2/2 (100%) |  |

The data indicate the number (%)
